# Beta-catenin inhibits bovine parainfluenza virus type 3 replication via innate immunity pathway

**DOI:** 10.1186/s12917-020-02291-w

**Published:** 2020-03-03

**Authors:** Xinying Du, Wenqi He, Hongbin He, Hongmei Wang

**Affiliations:** 1 Ruminant Diseases Research Center, College of Life Sciences, Shandong Normal University, Jinan, 250014 China; 2 College of Veterinary Medicine, Jilin University, 5333 Xi’an Road, Changchun, 130062 China

**Keywords:** Bovine parainfluenza virus type 3 (BPIV3), β-Catenin, Wnt/β-catenin signal pathway, GSK3β, Viral replication

## Abstract

**Background:**

Bovine parainfluenza virus type 3 (BPIV3) is one of the important viral respiratory agents associated with the bovine respiratory disease complex (BRDC) in cattle. Previous study has demonstrated that infection of BPIV3 causes innate immune response within the host cell. β-catenin is a key component of the Wnt/β-catenin signal pathway which is involved in the regulation of interferon-beta (IFN-β) transcription. Some viruses can activate while others can inhibit the Wnt/β-catenin signaling pathway. However, the role of β-catenin in BPIV3 infection remains unclear.

**Results:**

Here we found that the expression of β-catenin mRNA was up-regulated and β-catenin protein was down-regulated after BPIV3 infection in MDBK cells. Moreover, it was confirmed that overexpression of β-catenin suppressed BPIV3 replication and knockdown of β-catenin promoted viral replication, suggesting that β-catenin inhibits BPIV3 replication. Furthermore, IFN-β signal pathway and virus titer analysis using the GSK3β inhibitor (LiCl) revealed that Wnt/β-catenin can serve as a mechanism to suppress virus replication in infected cells. The results indicated that LiCl promoted the expression and accumulation in the nucleus of β-catenin, which further promoted the expression of IFN-β and OSA1 and suppressed BPIV3 replication. Most importantly, BPIV3 down-regulating β-catenin protein expression was due to degradation of GSK3β mediated proteasome pathway.

**Conclusions:**

In summary, we discovered the relationship between β-catenin and BPIV3 replication. These results provided further insight into the study of BPIV3 pathogenesis.

## Background

Bovine parainfluenza virus type 3 (BPIV3), also known as Bovine respirovirus 3, belongs to the Paramyxoviridae Respirovirus genus. BPIV3 is a single-strand negative-sense RNA virus that enveloped and non-segmented [1]. The BPIV3 genome encodes six structural proteins including the nucleocapsid (N), phosphoprotein (P), matrix (M), fusion (F), hemagglutinin-neuraminidase (HN), and large (L) proteins. Three accessory proteins are encoded by the P gene, including the C, V and D proteins [2]. BPIV3 has demonstrated strong perniciousness in regards to both adult and young cattle, and identified one of the viral respiratory agents that caused serious economic losses in cattle [3]. In addition, together with other viruses and pathogens, BPIV3 can cause complications and form BRDC with symptoms such as cough, fever and nasal discharge which has become a major problem affecting the health of cattle worldwide and a major risk factor for animal husbandry [4].

Protecting host cells from virus invasion and infection is an important role of antiviral innate immunity [5, 6]. During viral infection, the term includes a variety of signaling pathways that regulate the expression of a range of genes to eliminate invading viruses and control inflammation [7–9]. Among these signaling pathways, the Wnt/β-catenin pathway has been demonstrated to play an important role in the activation of IFN-β and inflammatory factors during virus infections. IFN-β and inflammatory factors play pivotal roles in the innate immune response [10, 11].

Wnt/β-catenin signaling pathway is not only an important way to regulate the early development of embryonic, maintain tissue homeostasis in adults and central nervous system regeneration, but also closely related to cell proliferation, differentiation, migration and apoptosis [12–14]. The abnormal regulation of Wnt/β-catenin signal is closely related to many diseases [15–17]. In the absence of Wnt ligands, the Wnt/β-catenin signaling pathway is in a “ off “ state, and β-catenin is at a low level under the control of ubiquitin proteasome system [18]. β-catenin in the cytoplasm is found within a degradation complex associated with adenomatous polyposis coli (APC), axonal protein (AXIN), casein kinase1α (CK1α), glycogen synthase kinase-3β (GSK3β) and the E3-ubiquitin ligase-β-TrCP. CK1α and GSK-3β successively phosphorylated β-catenin at specific sites [19]. Phosphorylated β-catenin is recognized and ubiquitinated by β-TrCP, subsequently degraded by proteasome. β-catenin is thereby maintained at a low level in the cytoplasm. When the Wnt signal is present, Wnt/β-catenin signal pathway is at “on” state. The degradation complex APC/AXIN/CK1α/GSK3β is dissociated, and β-catenin in the cytoplasm remains stable and gradually accumulates and enters into the cell nucleus [19]. Subsequently, the β-catenin in the nucleus binds to TCF/LEF and initiates the transcription of downstream genes such as IFN-β, c-myc and cyclin D1 [20–22]. Therefore, β-catenin is a key signal transduction factor in Wnt/β-catenin signaling pathway [23], and β-catenin is tightly regulated at three hierarchical levels: transcriptional activity, protein stability, and subcellular localization [18].

Several studies have showed the interaction of the Wnt/β-catenin signaling pathway with viruses, such as the human immunodeficiency virus (HIV), the porcine circovirus-like virus P1, the Human Cytomegalovirus (HCMV), the hepatitis C virus (HCV) and so on. Some viruses can activate while others deactivate the Wnt/β-catenin signaling pathway. For example, HIV suppresses Wnt/β-catenin signaling transduction by interacting its viral negative regulatory factor with β-catenin [24], HCMV infection can inhibit Wnt/β-catenin transcriptional activity [25]. On the contrary, the HCV NS5A protein can activate the Wnt/β-catenin signaling pathway [26]. Moreover, Wnt/β-catenin signaling pathway also has different effects on different viruses, activation of Wnt/β-catenin signaling pathway can promote or inhibit virus replication. For instance, the Wnt/β-catenin signaling pathway stimulates the productive infection of bovine herpesvirus 1 [27]. Beyond that, Wnt3a activates Wnt/β-catenin pathway to increase influenza virus mRNA and virus production [23, 28]. Otherwise, knocking down β-catenin enhances the transcription of HIV [29], and activation of Wnt/β-catenin by LiCl treatment inhibits the proliferation of HIV in peripheral mononuclear cells [30]. From these we can see that Wnt/β-catenin participates in many viral infections through complex and diverse mechanism, but its role in BPIV3 infection remains unclear.

In this study, we first investigated the effect of Wnt/β-catenin during BPIV3 infection, and found that the expression of β-catenin mRNA and protein levels was up-regulated and down-regulated respectively during BPIV3 infection. BPIV3 replication was also either suppressed or promoted with overexpression or knockdown of β-catenin gene. Furthermore, under the effect of GSK3β inhibitor LiCl, the expression and accumulation of β-catenin in the nucleus were promoted, and the expressions of IFN-β and OSA1 were further promoted to suppress BPIV3 replication. Further studies revealed that BPIV3 down-regulated β-catenin protein expression due to degradation of GSK3β mediated proteasome pathway. Our findings largely provide some novel insights into virus and host interactions during BPIV3 infection.

## Results

### BPIV3 infection regulated the expression of β-catenin

**Fig. 1:**
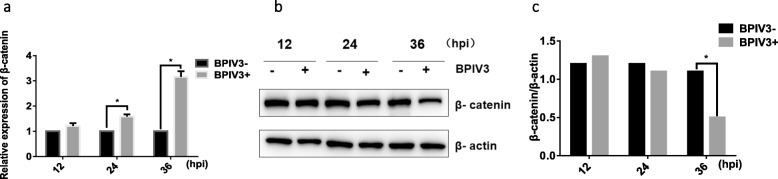
BPIV3 infection regulated the expression of β-catenin **a** qRT-PCR analyses the expression of β-catenin. Expression of β-catenin gene in MDBK cells infected with BPIV3 (1MOI) at 12, 24, and 36 h was assessed by qRT-PCR and β-actin was used as internal control. Mock-infected cells were used as negative control. The results represent the average of three biological replicates. Error bars represent SD (*, *P* < 0.05; **, *P* < 0.01). **b** Western blot analyses β-catenin protein expression. The expression of β-catenin protein in MDBK cells infected with BPIV3 at a MOI of 1 or mock infection at 12, 24 and 36 h was assessed by western blot with anti-β-catenin antibody, and β-actin was used as an internal control. Western blot represents one of three independent experiments. **c** β-catenin protein levels were quantitated by density values using Image J software

To explore whether BPIV3 infection affected the expression of β-catenin, Madin-Darby Bovine Kidney Cells (MDBK) were infected with BPIV3 (1MOI) at different time points, the level of β-catenin mRNA was determined by qRT-PCR and the expression of β-catenin protein was detected by western blot. The results showed that the level of transcription of β-catenin gene significantly increased at different time points after BPIV3 infection compared to the control group (Fig. 1a). However, dramatically decreased expression level of β-catenin protein was observed in BPIV3 infected MDBK cells at 36 h compared to the control group (Fig. 1b). ImageJ software was used to analyze the results of Fig. 1b, and the result showed in Fig. 1c was further illustrated by quantitative statistics. Thus, the expression of β-catenin protein was down-regulated, demonstrating that BPIV3 infection can affect the expression of β-catenin at post translation level.

### β-Catenin inhibited BPIV3 replication

**Fig. 2:**
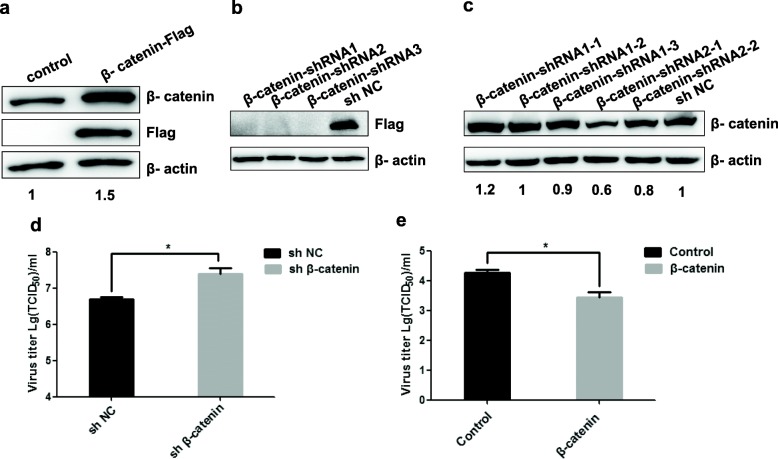
β-catenin inhibited BPIV3 replication in MDBK cells (**a**) The expression level of β-catenin protein in stable expressing cell lines was detected by western blot with anti-β-catenin antibody, anti-Flag antibody, and β-actin was used as an internal standard. Numbers below the image was β-catenin/β-actin ratios of band optical density values from Image J software. **b** The knockdown efficiency of β-catenin-shRNA recombinant vector in 293 T cells with anti-Flag antibody. **c** The screening about knockdown of β-catenin of MDBK cell lines, β-catenin-shRNA-2-1 was the effective silencing β-catenin cell lines, and named β-catenin-shRNA. Numbers below the image was β-catenin/β-actin ratios of band optical density values. **d** Effect of β-catenin silencing on BPIV3 replication. β-catenin knockdown cell lines and control cell lines were infected with BPIV3 (0.1 MOI) for 24 h, then the titers of BPIV3 infected cells were determined by TCID_50_ assay. The data were means with SD from four independent experiments (*, *P* < 0.05). **e** Effect of β-catenin overexpressing on BPIV3 replication. MDBK cells stably expressing β-catenin was infected BPIV3 (0.1 MOI) for 24 h, and cells were collected at 24 h and repeated freezing and thawing 3 times. Virus titers determined by the Reed-Muench endpoint method and expressed as log10 tissue culture infections dose 50 (TCID_50_) per milliliter. The results represent the average of three independent experiments (*, *P* < 0.05)

To further investigate the role of β-catenin in BPIV3 infection, MDBK cell lines expressing the Flag epitope-tagged β-catenin were constructed, and the expression of β-catenin protein probed with anti-Flag antibodies and anti-β-catenin antibodies was analyzed by western blot respectively. A significant up-regulation of β-catenin expression was observed in stable cell lines compared to empty vector expressed cell lines (Fig. 2a), suggesting that the cell lines with ectopic stable expression of β-catenin gene were successfully established. Subsequently, overexpression of β-catenin cells were infected with BPIV3 at a MOI of 0.1 for 24 h and viral titers were determined by the 50% tissue culture infected dose (TCID_50_). It was found that the amounts of infectious virus at 24 h decreased at about 8-fold in β-catenin-overexpression cells compared to control group cells (Fig. 2e).

To further explore the effect of β-catenin on BPIV3 replication reversely, the shRNAs targeting bovine β-catenin were designed. In order to verify the interference efficiencies, pLVX-IRES-Flag-β-catenin with shRNA1/2/3 or shNC were co-transfected into 293 T cells, respectively. As shown in Fig. 2b, the interference efficiencies of these three shRNAs were very well. Subsequently, β-catenin-shRNA1 and β-catenin-shRNA2 were selected to constructed MDBK cell lines with knockdown of β-catenin gene by recombinant lentivirus. The western blot analysis was performed to identify the efficiency of endogenous β-catenin knockdown (Fig. 2c). From the results, we concluded that the expression of β-catenin protein was effectively inhibited in β-catenin-shRNA-2-1 cell lines. Similarly, these β-catenin-knockdown cells were infected with 0.1 MOI BPIV3 for 24 h and viral titers were assayed. As the results showed in Fig. 2d, knockdown of β-catenin in MDBK cells resulted in increased viral yields compared with shNC cells. Together with that overexpression of β-catenin decreased the viral titers, these results showed that β-catenin inhibited BPIV3 replication in MDBK cells.

### β-Catenin inhibited BPIV3 replication via strengthening IFN-β expression

**Fig. 3:**
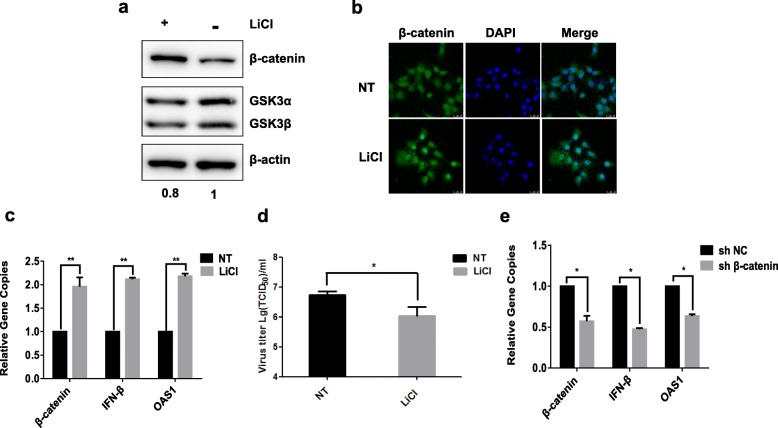
β-catenin inhibited BPIV3 replication via strengthening IFN-β expression (**a**) The expression level of β-catenin and GSK3β in MDBK cells treated or not treated by LiCl at 20 mM for 24 h was assayed by western blot with anti-β-catenin antibody, anti-GSK3β antibody, and β-actin was used as an internal standard. Numbers below the image was GSK3β/β-actin ratios of band optical density values from Image J software. **b** The cell location of β-catenin was detected by immunofluorescence. MDBK was treated by LiCl (20 mM) or not treated (NT) for 24 h were labeled with an anti-β-catenin antibody, secondary antibodies, and an agent to visualize the nucleus (Hoechst). The green indicates β-catenin while the blue indicates nucleus. **c** IFN-β and OAS1 genes in MDBK cells treated or not treated by LiCl were detected using qRT-PCR (*, *P* < 0.05; **, *P* < 0.01). **d** MDBK cells were treated or not treated by LiCl at 20 mM for 24 h with BPIV3 infection at 0.1MOI, then the titers of BPIV3 infected cells were determined by TCID_50_ assay. **e** Cells with knockdown of β-catenin and shNC cells were infected by BPIV3, then the mRNA of IFN-β and OAS1 were analyzed by qRT-PCR (*, *P* < 0.05; **, *P* < 0.01)

Previous studies have shown that as a transcription factor, β-catenin up-regulated the expression of IFN-β through T-cell factor (TCF) binding sites after entering the nucleus [22]. Given that our results demonstrated how the overexpression of β-catenin gene suppressed BPIV3 replication in MBDK cells, we further evaluated whether the reduction of BPIV3 proliferation with the overexpression of β-catenin was a result by facilitations of IFN-β expression. LiCl, a potential inhibitor of GSK3β was used as a tool to up-regulate β-catenin in MDBK cells. As expected, GSK3β was down-regulated after treated by LiCl (20 mM) for 24 h (Fig. 3a), which indicated that the activity of GSK3β was inhibited. As shown in Fig. 3b, β-catenin was scarcely distributed in both cell cytoplasm and cell nucleus in normal MDBK cells. However, the expression of β-catenin in the cell nucleus was significantly enhanced after the treatment by LiCl for 24 h compared to those that were not treated. As the expression of β-catenin increased, the blue color in the nucleus became lighter and the white color became deeper when merged. To verify the expression of downstream genes, IFN-β and one of its major interferon (IFN)-stimulated genes (ISGs), OAS1 were examined by using qRT-PCR. The results were shown in Fig. 3c, the expressions of β-catenin, IFN-β and OAS1 were all up-regulated after treated by LiCl. Furthermore, the titer of BPIV3 was significantly reduced after treated by LiCl (Fig. 3d). These results suggested that β-catenin accumulated in the cytoplasm and then entered the nucleus to enhance IFN-β and ISGs expression, which helped to inhibit BPIV3 replication.

In order to verify the effect of β-catenin on IFN-β expression in reverse, post-infection results in β-catenin-knockdown cells were also monitored. As shown in Fig. 3e, β-catenin was significantly decreased in cells with knockdown of β-catenin compared to shNC cells, while the amount of IFN-β and OAS1 were decreased correspondingly. Taken together, it was indicated that β-catenin inhibited BPIV3 infection via activation of the innate immune signaling pathway encompassing IFN-β and OAS1.

### BPIV3 infection induced degradation of β-catenin via GSK3β

**Fig. 4:**
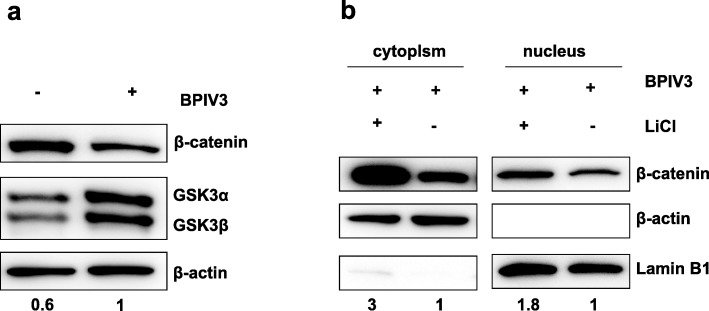
BPIV3 infection induced degradation of β-catenin via GSK3β (**a**) MDBK cells were infected or mock infected by BPIV3 at 1MOI for 36 h, and were subsequently harvested for western blot to detect GSK3β expression and β-catenin expression. Numbers below the image was GSK3β/β-actin ratios of band optical density values from Image J software. **b** Nucleus and cytoplasm were separated to detect the expression of β-catenin. MDBK cells were pretreated or not with LiCl of 20 mM for 24 h, and then were infected by BPIV3 at 1 MOI for 24 h. Subsequently, cells were harvested for nucleus and cytoplasm separation and western blot analysis. β-actin and Lamin B1 were used as an internal standard of cytoplasmic protein and nucleus protein, respectively. Numbers below the image was β-catenin/β-actin or β-catenin/Lamin B1 ratios of band optical density values

Given that our results showed significant post-BPIV3-infection decrease in β-catenin’s expression at 36 h (Fig. 1), it is highly suspicious that post-transcription regulation mechanisms of β-catenin might exist for the BPIV3 invasion. We thus monitored the change in GSK3β, a known component in the degradation complexes of β-catenin, in MDBK cells that were infected with BPIV3 (1 MOI) for 36 h. The result showed that the expression of GSK3β was up-regulated post-infection. This would partially explain why β-catenin expression was reduced after BPIV3 infection (Fig. 4a). We then tested this relationship in reverse and added LiCl to pretreat MDBK cells for 24 h. The β-catenin protein levels were monitored with a nucleus and cytoplasm separation after 24 h of infection by BPIV3 (1 MOI). It showed that the β-catenin expression both in cell nucleus and cell cytoplasm all increased after treated by LiCl treatment comparing to the cells that not treated (Fig. 4b). This illustrated that although the expression of β-catenin can be down-regulated when MDBK cells were infected by BPIV3 solely, the activity of GSK3β can be inhibited after pretreatment with LiCl. MDBK cells were infected by BPIV3 after pretreated with LiCl, the down-regulation effect of BPIV3 on β-catenin can also be partially relieved. Together, it can be assured that degradation of β-catenin via GSK3β occurred after BPIV3 infection.

## Discussion

β-catenin is a key component in the activation of Wnt/β-catenin pathway and thus plays an important role in antiviral immune response. It can directly or indirectly inhibit the replication of HIV, HCMV, HCV and other types of viruses or control their pathogenicity [24–26]. However, whether β-catenin directly interferes with the replication of BPIV3 requires further and deeper inquiries. Our studies confirmed that after the infection of BPIV3 in MDBK cells, there’s been up-regulation of β-catenin’s mRNA expression (Fig. 1a), and that the protein level of β-catenin significantly decreased 36 h post-infection (Fig. 1b and c). These data suggested that the infection of BPIV3 instigated the expression of β-catenin mRNA and in addition affected the cellular β-catenin accumulation level. This may suggest us that BPIV3 infection affected the expression of β-catenin after transcription, the increasing of β-catenin mRNA and the decreasing of β-catenin protein were regulated by different mechanisms. Most importantly, experiments utilizing the β-catenin overexpressed cell lines demonstrated significant inhibition of viral replication by an increased level of β-catenin; experiments utilizing β-catenin knockdown cells showed that the replication of the virus within these cells has been augmented. Together these data supported the antiviral function of β-catenin during the infection of BPIV3 (Fig. 2).

Specifically, in the activated Wnt/β-catenin pathway, the degradation complex of β-catenin, AXIN/APC/GSK3β/CK1 complex is depolymerized [17]. β-catenin aggregates within the plasma and enters the nucleus to combine with TCF/LEF, which in turn activates the transcription of down-stream genes. It is known that LiCl can inactivate GSK-3β and activate β-catenin signaling pathway [30]. In this study, we treated MDBK cells with LiCl and found that the expression of GSK3β was inhibited and the expression of β-catenin was up-regulated (Fig. 3a), facilitating the nucleic accumulation (Fig. 3b). Meanwhile, the expression of IFN-β and downstream anti-viral gene OAS1 has also increased in BPIV3 infected cells pretreated by LiCl (Fig. 3c), which significantly inhibited the replication of the virus (Fig. 3d). It appears that LiCl activates the Wnt/β-catenin signaling pathway and in turn prompts the expression of IFN-β and downstream antiviral genes, this significantly inhibits viral replication. In addition, β-catenin silenced cell lines have demonstrated down-regulation of the mRNA expression of β-catenin, IFN-β and OAS1 after being infected by BPIV3. This indicates that β-catenin, as a transcription factor, deactivates the transcription of IFN-β and therefore influences the expression of downstream antiviral genes. It is a finding that corroborates the results in researches on β-catenin’s suppression of newcastle disease virus and rift valley fever virus [22].

Many other viral infections have been shown to correlate with the functionality of β-catenin. For example, both Porcine Circovirus-like virus and Human Cytomegalovirus inhibit the classical Wnt signal pathway post-infection [8, 31]. When Wnt/β-catenin pathway is at the deactivation stage, the protein kinase GSK3β phosphorylates serine at site 33 and 37 on β-catenin [17]. The phosphorylated product would then react with E3 ligase to undergo the process of ubiquitination and eventually be degraded by protease to keep the concentration of β-catenin in the plasma at a low level. In our studies we observed that β-catenin level has decreased in MDBK cells after the infection of BPIV3. We then deduced and experimentally tested whether this decrease has something to do with the GSK3β mediated proteasome degradation. The result was that the level of GSK3β increased 36 h after BPIV3 infection. If cells were treated with GSK3β repressor LiCl and then infected, the repressive function of LiCl would cause plasmatic β-catenin to accumulate and enter the nucleus (Fig. 4). These results indicate that BPIV3 infection can induce the expression of GSK3β and that GSK3β mediate the degradation of β-catenin. The finding supports and certifies the theorized pathway of GSK3β mediated phosphorylation-ubiquitination-proteasome degradation of β-catenin.

**Fig. 5:**
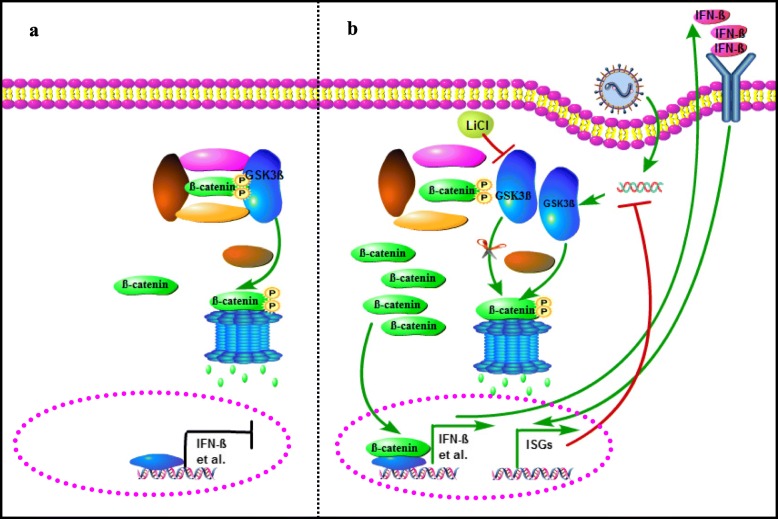
The model that β-catenin inhibits BPIV3 infection via innate immunity pathway (**a**) The inactivated Wnt/β-catenin pathway. β-catenin is constantly phosphorylated by GSK3β, and its phosphorylations constitute a signal for β-catenin ploybiquitination and hydrolysis by proteasome. **b** The proposed antiviral response of the β-catenin pathway. On the one hand, BPIV3 infection would up-regulate GSK3β to facilitate the degradation of β-catenin. On the other hand, the treatment of LiCl would inhibit GSK3β to prevent β-catenin degradation, and accumulated β-catenin could enter the nucleus to up-regulate expression of IFN-β, which then instigates transcription of downstream ISGs, and ISGs could inhibit the proliferation of the BPIV3

Multiple studies have shown that different virus may employ different mechanisms to manipulate the process of Wnt/β-catenin pathway’s participation in viral replication [20, 21, 31]. In this study, β-catenin up-regulated the expression of IFN-β and ISG, inhibited BPIV3 proliferation. The expression of GSK3β was up-regulated by BPIV3 infection, and suppressed by LiCl as a specific inhibitor of GSK3β. BPIV3 infection down-regulated the expression of β-catenin via GSK3β mediated proteasome degradation. A model explaining these observations is shown in Fig. 5. Although detailed mechanisms remain to be clarified, these examples showed that β-catenin has been replaced by viruses at different stages of its life cycle, and possibly through different mechanisms. Since viral infections usually sensitize various signaling pathways, higher chance arises that these pathways would interact with each another in a multitude of ways, either facilitating or antagonizing one another [32–34]. Therefore, studying about the activation mechanism of viral infection upon the Wnt/β-catenin pathway and the interdependent relationships between Wnt/β-catenin pathway and other pathways would help us to further understand viral pathology. Clinical application against BPIV3 infection, small molecules against BPIV3 infection would be screened to inhibit GSK3β activity or up-regulate β-catenin protein. It would provide new targets for the diagnosis and treatment of infectious diseases.

## Conclusion

In summary, results of our study indicate that β-catenin plays a significant role in BPIV3 infection. BPIV3 infection suppresses the protein level of β-catenin, since β-catenin can affect viral replication by promoting the expression of IFN-β and ISGs. Moreover, viral invasion mostly down-regulates the amount of β-catenin by hijacking its GSK3β mediated proteasome degradation. Overall, our study showed the important effect of β-catenin on BPIV3 infection and prompts us to do more in the future to reveal its comprehensive involvement in virus-host interaction.

## Methods

### Virus and cell lines

BPIV3 strain was isolated and stored in the Ruminant Disease Research Center, Shandong Normal University, Jinan, Shandong Province, China. TCID_50_ of BPIV3 was determined by the Reed-Muench method, which was described previously [35, 36].

The 293 T (GDC0067) originating from China Center for Type Culture Collection (CCTCC) and MDBK cell were provided by American Type Culture Collection (ATCC No.: CCL-22). MDBK cells and 293 T cells were preserved in the Ruminant Disease Research Center, Shandong Normal University, and cultured in Dulbecco’s Modified Eagle’s Medium (DMEM, Invitrogen, Carlsbad, CA, USA) and supplemented with 10% fetal bovine serum (FBS, Gibco, USA) at 37 °C in a humidified, 5% CO_2_ atmosphere.

### Antibodies

Anti-β-catenin antibody from Santa Cruz Biotechnology (catalog number sc-7963) was used for immunofluorescence and western blot. Anti-β-actin, anti-Lamin B1, anti-GSK3, and anti-Flag antibodies from Santa Cruz Biotechnology (catalog number sc-69879, sc-377000, sc-7291, sc-166355) were used for western blot. The secondary antibodies used for immunofluorescence were FITC-conjugated goat anti-mouse IgG (catalog number ab6785) from Abcam. The secondary antibodies used for western blot were HRP-linked mouse IgG (sc-516,102) from Santa Cruz Biotechnology.

### Quantification of deferentially expressed β-catenin, IFN-β, OAS1 by qRT-PCR

MDBK cells were infected with BPIV3 at a MOI of 1, and total cells were collected at 12 h, 24 h and 36 h post infection to detect the differential expression of β-catenin using specific primers as shown in Table 1. To further study how β-catenin inhibiting BPIV3 replication, MDBK cells were directly treated with 20 mM LiCl added to DMEM, and the cells were collected at 24 h post reaction were subjected to detect the differential expressions of β-catenin, IFN-β, and OAS1. RNA extraction was performed by using Cell Total RNA Isolation Kit (Foregene Biotech Co., Ltd., Chengdu, China). The concentration of RNA was measured with a NanoDrop ND-2000 spectrophotometer (Thermo Fisher Scientific, Waltham, MA). Quantification of β-catenin, IFN-β and OAS1 gene expression was carried out using One Step TB Green® PrimeScript™ RT-PCR Kit (TaKaRa, Dalian, China) according to the manufacturer’s protocols on Roche 96 Real Time PCR System (Roche Applied Science, Germany) with the specific primers as shown in Table 1. The mRNA expression levels were analyzed according to delta-delta CT method (2^-△△Ct^), and β-actin mRNA expression levels were used for normalization process [35].

**Table 1:** List of primers used in this study

Primer name	Sequence (5′-3′)	Application
β-catenin-F	ACTGCTGGGACCTTGCACAA	qRT-PCR to detect β-catenin
β-catenin-R	ACTGGTGAACCGAGCATCTTCA	
IFN-β-F	CCTGTGCCTGATTTCATCATGA	qRT-PCR to detect IFN-β
IFN-β-R	GCAAGCTGTAGCTCCTGGAAAG	
OAS1-F	GATGTCCTGCCCGCCTTT	qRT-PCR to detect OAS1
OAS1-R	GCTGGACGTAGATTTGAGGGTTA	
β-actin-F	GATGAGATTGGCATGGCTTTA	qRT-PCR to detect β-actin
β-actin-R	AACCGACTGCTGTCACCTTC	
β-catenin-F (*Xba I*)	GCTCTAGA*GCCACC*ATG**GATTACAAGGATGACGACGATAAG**ATGGCTACCCAAGCTGAT	PLVX-IRES-β-catenin-puro
β-catenin-R (*BamH I*)	CGGGATCCCGTTACAGGTCAGTATCAAA	
β-catenin-shRNA1-F	GATCCGGATGTGGATACCACCCAAGTCTCGAGACTTGGGTGGTATCCACATCCTTTTTG	β-catenin-shRNA1
β-catenin-shRNA1-R	AATTCAAAAAGGATGTGGATACCACCCAAGTCTCGAGACTTGGGTGGTATCCACATCCG	
β-catenin-shRNA2-F	GATCCGCACAATCTTTCCCACCATCGCTCGAGCGATGGTGGGAAAGATTGTGCTTTTTG	β-catenin-shRNA2
β-catenin-shRNA2-R	AATTCAAAAAGCACAATCTTTCCCACCATCGCTCGAGCGATGGTGGGAAAGATTGTGCG	
β-catenin-shRNA3-F	GATCCGCTTATGGCAATCAAGAAAGCCTCGAGGCTTTCTTGATTGCCATAAGCTTTTTG	β-catenin-shRNA3
β-catenin-shRNA3-R	AATTCAAAAAGCTTATGGCAATCAAGAAAGCCTCGAGGCTTTCTTGATTGCCATAAGCG	

Note: Underline, underline stands for the restriction endonuclease enzyme cutting sites, Italic, italic stands for kozaka sequence, Bold, blod stands for Flag-tag sequence

### Construction of overexpression and silence recombinant lentiviral plasmids of β-catenin

The PCR primer β-catenin-F (*Xba I*) and β-catenin-R (*BamH I*) shown in Table 1 were used to construct the overexpression recombinant vector pLVX-IRES-Flag-β-catenin. The overexpression recombinant vector and pLVX-IRES-puro vector were transfected into 293 T cells, respectively. After transfection 48 h, the expression of β-catenin was identified by western blot with anti-Flag antibody. In order to stabilize the knockdown of endogenous β-catenin expression in MDBK cells, the silence recombinant lentiviral vectors for delivering short hairpin RNA (shRNA) targeting β-catenin gene was constructed according to an established method [37]. According to the sequence of bovine β-catenin in the GenBank (NM_001076141), three couples of small interfering RNA sequences (Table 1) were designed by the web browser BLOCK-iT™ RNAi Designer (https://rnaidesigner.thermofisher.com/ rnaiexpress/design.do), synthesized by TSINGKE Biological Technology Co., Ltd. (Beijing, China) and cloned into the pYr-Lvsh lentiviral vector and named β-catenin-shRNA1/2/3, respectively [38]. The pYr-Lvsh vector was named shNC. The silence recombinant lentiviral vectors or shNC with pLVX-IRES-Flag-β-catenin vector were co-transfected into 293 T cells, respectively. After 48 h, the transfected cells were identified by western blot with anti-Flag antibodies to select the recombinant vector with effective silent β-catenin gene.

### Lentiviral packaging and stable cell lines of β-catenin overexpression and knockdown

The overexpression and silence recombinant vectors of β-catenin and four plasmid-based lentiviral packaging system pLP1, pLP2, pLP/VSVG were co-transfected into 293 T cells for packaging recombinant lentivirus, respectively. The cellular extraction was harvested 48 h after co-transfection and was then used to infect MDBK cells for 48 h, respectively. The resistant colonies were obtained by incubation with puromycin of 5 μg/mL in DMEM for 10 d. The resistant colonies were identified by western blot with anti-Flag antibody and anti-β-catenin antibody, respectively.

### Measurement of BPIV3 replication in MDBK cells

MDBK cells with stable overexpression or knockdown of β-catenin and control MDBK cells were infected with BPIV3 at 0.1 MOI for 24 h. Cells and culture medium were repeatedly frozen and thawed three times to determine the replication level of BPIV3. MBDK cells were passaged in 96-well plates in DMEM with 10% fetal calf serum, and infected with 10-fold dilutions of BPIV3 harvested above. After 48 h, viral cytopathic effect was monitored and TCID_50_ of virus was calculated using the Reed–Muench method.

### Nucleus and cytoplasm separation experiment

MDBK cells were pretreated by LiCl at a final concentration of 20 mM for 24 h, and then were infected by 1 MOI BPIV3 for 24 h. After that, cells were harvested and the nucleus and cytoplasm were separated according to the manufacturer’s protocol of Minute™ Cytoplasmic and Nuclear Fractionation kit (Invent Biotechnologies, Inc) and then subjected to western blot analysis. β-actin and Lamin B1 were used as internal references of cytoplasmic and nucleus protein, respectively.

### Western blot analysis

MDBK cells infected by BPIV3 or pretreated by LiCl or not were subjected to 10% SDS gel electrophoresis and transferred onto PVDF membranes (Epizyme scientific, Shanghai, China). The membranes were then blocked using 8% non-fat dry milk dissolving in TBST for 2 h, incubated with primary antibodies at a dilution of 1:2000 for 2 h at room temperature. Afterwards the membranes were washed by TBST and incubated with secondary antibodies for 1 h at room temperature. Finally, P-ECL Chemiluminescence Kit (Epizyme scientific, Shanghai, China) was used to show the target protein band using chemiluminescence imaging system (Tanon Science & Technology Co., Ltd., Shanghai, China).

### Immunofluorescence

MDBK cells in good condition were dissolved to form a cell suspension and seeded in 24-well plates containing the coverslips. When cells reached 50% confluence in the coverslips, LiCl at 20 mM was added for 24 h. Next, the original culture was discarded, MDBK cells were washed with phosphate-buffered saline (PBS), fixed with 4% paraformaldehyde (PFA, VETEC™, Shanghai, China)/PBS for 15 min at room temperature, washed with PBS and permeabilized with 1% Triton X-100 (Biofroxx, Germany)/PBS for 10 min. Subsequently, cells were blocked with 0.5% bovine serum albumin (BSA, VETEC™, Shanghai, China), incubated with the corresponding primary antibodies diluted in BSA for 1 h at room temperature. Cells were then washed with PBS and incubated with the corresponding secondary antibodies for 1 h at room temperature. After the 4′,6-diamidino-2-phenylindole (Beyotime, China) was added to dye nucleus for 5 min without light, cells were observed with a confocal microscope and recorded.

### Statistical analyses

Graphpad prism software (version 5.0; San Diego, CA, USA) was used to perform statistical analysis. Results were presented as the mean values ± standard deviation (SD) from at least three independent experiments, and *P* value below 0.05 was considered statistically significant (*P < 0.05; **P < 0.01).

## Data Availability

The datasets used and/or analysed during the current study are available from the corresponding author on reasonable request.

## Abbreviations

APC: Adenomatous polyposis coli
AXIN: Axonal protein
BPIV3: Bovine parainfluenza virus type 3
BRDC: Bovine respiratory disease complex
BSA: Bovine serum albumin
CK1α: Casein kinase1α
DMEM: Dulbecco’s modified Eagle’s medium
FBS: Fetal bovine serum
GSK3β: Glycogen synthase kinase-3β
IFN-β: Interferon-beta
ISG: Interferon-stimulated gene
LiCl: Lithium chloride
MDBK: Madin-Darby Bovine Kidney
PBS: Phosphate-buffered saline
qRT-PCR: Quantitative reverse transcription polymerase chain reaction
TCID_50_: 50% tissue culture infected dose
